# Multilocus marker-based delimitation of *Salicornia persica* and its population discrimination assisted by supervised machine learning approach

**DOI:** 10.1371/journal.pone.0270463

**Published:** 2022-07-27

**Authors:** Rahul Jamdade, Khawla Al-Shaer, Mariam Al-Sallani, Eman Al-Harthi, Tamer Mahmoud, Sanjay Gairola, Hatem A. Shabana

**Affiliations:** 1 Sharjah Seed Bank and Herbarium, Environment and Protected Areas Authority (EPAA), Sharjah, United Arab Emirates; 2 Nature Conservation Sector, Egyptian Environmental Affairs Agency, Cairo, Egypt; Institute for Biological Research, University of Belgrade, SERBIA

## Abstract

The *Salicornia* L. has been considered one of the most taxonomically challenging genera due to high morphological plasticity, intergradation between related species, and lack of diagnostic features in preserved herbarium specimens. In the United Arab Emirates (UAE), only one species of this genus, *Salicornia europaea*, has been reported, though investigating its identity at the molecular level has not yet been undertaken. Moreover, based on growth form and morphology variation between the Ras-Al-Khaimah (RAK) population and the Umm-Al-Quwain (UAQ) population, we suspect the presence of different species or morphotypes. The present study aimed to initially perform species identification using multilocus DNA barcode markers from chloroplast DNA (cpDNA) and nuclear ribosomal DNA (nrDNA), followed by the genetic divergence between two populations (RAK and UAQ) belonging to two different coastal localities in the UAE. The analysis resulted in high-quality multilocus barcode sequences subjected to species discrimination through the unsupervised OTU picking and supervised learning methods. The ETS sequence data from our study sites had high identity with the previously reported sequences of *Salicornia persica* using NCBI blast and was further confirmed using OTU picking methods viz., TaxonDNAs Species identifier and Assemble Species by Automatic Partitioning (ASAP). Moreover, matK sequence data showed a non-monophyletic relationship, and significant discrimination between the two populations through alignment-based unsupervised OTU picking, alignment-free Co-Phylog, and alignment & alignment-free supervised learning approaches. Other markers viz., rbcL, trnH-psbA, ITS2, and ETS could not distinguish the two populations individually, though their combination with matK (cpDNA & cpDNA+nrDNA) showed enough population discrimination. However, the ITS2+ETS (nrDNA) exhibited much higher genetic divergence, further splitting both the populations into four haplotypes. Based on the observed morphology, genetic divergence, and the number of haplotypes predicted using the matK marker, it can be suggested that two distinct populations (RAK and UAQ) do exist. Further extensive morpho-taxonomic studies are required to determine the inter-population variability of *Salicornia* in the UAE. Altogether, our results suggest that *S*. *persica* is the species that grow in the present study area in UAE, and do not support previous treatments as *S*. *europaea*.

## Introduction

DNA barcoding has emerged as the most widely used tool assisting discrimination of taxonomically confusing plant species over the past few decades. The efficacy of various multilocus barcode markers has been demonstrated to resolve taxonomic ambiguities and achieve adequate species-level identification. However, in some cases, DNA barcodes do not provide enough resolution to identify plant species accurately, as the barcode region often overlaps among the sister taxa [1–4]. In such cases, where the barcode sequences do not show adequate resolution, the supplementary barcode regions could be required for species identification [5].

The morphology based-taxonomy has several limitations, such as morphologically similar species are often overlooked; even the available taxonomic keys for many species lack adequate identification characters required for the discrimination of immature plants [6]. Such issues could be addressed using molecular and genomic techniques, which are considered critical tools for genetic diversity analysis and accurate taxonomic identification to complement morphologically identified specimens [7].

In plants, a single universal barcode is not available to identify all plant groups [7–9]. Therefore, the data from a combination of barcode regions are used to identify all species in a particular class [10]. Researchers have suggested several different coding and non-coding barcode regions that are mainly located in the plastid or chloroplast genome; such barcodes are: matK, trnH-psbA,rbcL, atpF-atpH, rpoB, psbK-psbIr, and rpoC1 and others found in the ribosomal DNA of the nuclear genome which includes internal transcribed spacer 2 (ITS2) and external transcribed spacer (ETS) [6, 11–13]. However, ETS is considered more informative for phylogenetic reconstruction and allows better resolution of relationships than the ITS [14].

According to the analysis of The Plant Working Group (PWG) of the Consortium for the Barcode of Life (CBOL Plant Working Group 2009 [15]), the two markers maturase K (matK) and ribulose 1,5-bisphosphate carboxylase/oxygenase large subunit (rbcL) are efficient enough to discriminate plant taxa. The matK marker offers high-resolution potential but less universality, while the rbcL offers high universality but less species resolution [16]. Along with these core barcode markers, supplementary markers like ITS and trnH-psbA can further improve the species resolution [7, 9, 17]. Moreover, ETS has been used in many studies to achieve species-level identification [18, 19]. In *Salicornia*, the ETS has been used more effectively and is found to be informative at the genera level [20]. However, it has a low resolution at the species level [21]. Thus, researchers suggest a combination (concatenation) of multilocus DNA barcodes to classify and identify different plant species [11].

Along with selecting efficient barcode markers for species discrimination, it is essential to select the appropriate method for barcode analysis. Various conventional methods have been used for detecting the barcode gap, of which the ‘TaxonDNAs Species identifier’ [22] and recently developed ‘Assemble Species by Automatic Partitioning’ (ASAP) [23] are some of the most widely used methods; those depend on the alignment-based approach. In addition, alignment-free approaches, which include Co-phylog [24], Mash [25], and Multi-SpaM [26], have been utilized for the plant DNA sequence analysis [27]. Furthermore, supervised machine learning techniques have been extensively implemented that have demonstrated robustness in the discrimination of plant and animal taxa [28–32].

*Salicornia* L. (Salicornioideae, Amaranthaceae) is considered one of the most taxonomically challenging genera of angiosperms due to their intricate variation patterns [33]. *Salicornia’s* taxa require several characters to determine their identity, as they are not separated by one feature alone, often-showing intergradation between related species [34]. Furthermore, studies have suggested that the confusion in the delimitation of *Salicornia* taxa and morphological systematics in the Salicornioideae is mainly due to the reduced morphology combined with broad phenotypic plasticity (e.g., Slenzka et al. [35]; Piirainen [36]).

Traditionally, the genus *Salicornia* in UAE is represented by only one species *Salicornia europaea* [37–40]. Moreover, we have reported *Salicornia* sp. from Ras Al Khaimah (RAK) and Umm Al Quwain (UAQ) emirates during our field explorations. The plants from these two populations show ecological and life form differences. The plants at the RAK locality are biennial or annual and more often submerged in water than the plants at the UAQ population, which are annual. Besides, the plants from both populations have different forms. It is suspected that the plants in both populations may belong to another species or have different morphotypes of *Salicornia*. Therefore, we treated plant specimens separately from these populations according to the differences mentioned above. The present study aimed to determine the identity of *Salicornia* in studied populations in UAE and explore the genetic variation between these two populations using the chloroplast DNA (cpDNA) (rbcL, matK, and trnH-psbA) and nuclear ribosomal DNA (nrDNA) (ITS2 and ETS) barcode markers individually as well as using the concatenated approach, and to resolve species limits in collected accessions based on obtained molecular data.

## Materials and methods

### Ethics statement

For this research, the specimen collection was performed outside the Protected Areas (PAs), following the standard guidelines. The collected plant samples are not classified as endangered species.

### Study sites and sample collection

The first sampling locality in the emirate of Umm-Al-Quwain was at a north-facing (open water) coastal line of around 5 km length. In this area, samples were collected from *Salicornia* sp. growing on open tidal mudflats. The vegetation is dominated by the *Avicennia marina* followed by *Arthrocnemum macrostachyum*, *Suaeda vermiculata*, and *Halopeplis perfoliata*. The second sampling locality in the emirate of Ras-Al-Khaimah was at a south-facing tidal lagoon consisting of approximately 100 m in width. From the above two sites, the plant samples of *Salicornia* sp. were collected for analysis. The associated species were *A*. *macrostachyum*, *S*. *vermiculata*, and *Sesuvium portulacastrum*.

We collected a total of eighty specimens, forty from each population, covering the entire distribution of the sampled populations. Specimen vouchers collected from Umm-Al-Quwain (n = 40) and Ras-Al-Khaimah (n = 40) were deposited at the Sharjah seed bank and herbarium, Al Dhaid. Tissue samples were collected in liquid nitrogen and were preserved at -80° C until further analysis.

### DNA extraction

The tissue samples were ground to a fine powder in liquid nitrogen using a mortar and pestle. Genomic DNA extraction was then performed using the DNeasy Plant Mini Kit (Qiagen, Germany), as instructed by the manufacturer, with the necessary modifications. After adding the AP1 buffer and RNase A, the samples were incubated for about 3 hours on the heat block (Thermo Scientific—USA). Samples were eluted in Nuclease-Free Water. The isolated DNA was tested for its quality by gel electrophoresis (BioRad, USA) on a 1% agarose gel and quantity using spectrophotometric analysis (Denovix, USA).

### PCR amplification and purification

Three plastid barcode regions (rbcL, matK, and trnH-psbA) and the nuclear ribosomal barcode regions (Internal Transcribed Spacer (ITS) and External Transcribed Spacer (ETS)) were amplified via Polymerase Chain Reaction (PCR) (Biorad, USA and Applied Biosystems Veriti Thermal Cycler, USA) using forward and reverse primers of rbcL [41, 42], matK (proposed by Ki-Joong Kim, see [43]), trnH-psbA [44, 45], ITS2 [46, 47] and ETS [20] (S1 Table). The 25ul PCR reaction using a 5x FIREPol master mix was prepared to amplify the respective barcode region. Difficult samples were amplified using the KAPA3G plant PCR kit; it assists in amplicon recovery from the purified DNA and is efficient enough to perform direct PCR of plant samples [48]. PCR products were then verified through gel electrophoresis on a 2% agarose gel. Amplified products were purified using the MEGAquick-spinTM plus total fragment DNA Purification Kit (intron biotechnology, USA) and then sequenced commercially.

### Sequence analysis

Bidirectional sequencing was performed for rbcL, matK, trnH-psbA, ITS2, and ETS barcode markers. The obtained sequences were assembled and aligned in Geneious Prime v2020 (geneious.com) and MEGA X. [49], using the Muscle algorithm. The sequences were then submitted to NCBI GenBank through a web-based sequence submission tool ‘BankIt,’ and accessions numbers were obtained for all the studied barcode markers (rbcL: MW514466—MW514482, OM397125—OM397184; matK: MW514483—MW514497, OM397304—OM397363; trnH-psbA: MW514514—MW514530, OM397185—OM397244; ITS2: MW514498—MW514513, OM396936—OM396995 and ETS: MW514447—MW514465, OM397245—OM397303). Further, the sequences were subjected to the taxonomic evaluation using the NCBI GenBank BLASTn to obtain homologies between the fragments [50]. Along with the individual barcode marker dataset, concatenated datasets were prepared by combining the sequences using the alignment joiner in FaBox v1.5 [51]. The haplotypes and haplotype diversity were determined from these two datasets using DNASP v6.12 [52]. Further, unsupervised OTU picking methods TaxonDNAs Species identifier v1.8 [22] and ASAP (Assemble Species by Automatic Partitioning) [23] were employed. The species identifier was used to determine the percent identification through the ‘Best match (BM)’, ‘Best Closest match (BCM)’, and ’All species barcode’ criteria. ASAP was implemented through the web-server (bioinfo.mnhn.fr/abi/public/asap/asapweb.html) using the Jukes-Cantor (JC69) distance metric to determine the best partition representing the groups with the highest rate of identification. The phylogenetic analysis was done using MEGA X; initially, the model selection was performed based on the lowest AIC (Akaike information criterion) and BIC (Bayesian information criterion) scores. The phylogenetic tree was constructed using the maximum likelihood approach with the bootstrap support (of 1000). The tree was annotated in iTOL (Interactive Tree of Life) [53] using the OTUs identified through TaxonDNA and ASAP. For the population discrimination, along with the alignment-based analysis, the alignment-free analysis was performed to address the gaps and variable length of sequences using CAFÉ [54]. Moreover, the alignment-free genetic distances were calculated using the Co-Phylog algorithm. The obtained genetic distances were then used to generate population groups using ASAP’s JC69 metric.

Along with the unsupervised OTU picking methods, Supervised Machine Learning methods (SML) were implemented to recognize divergent taxa. The aligned datasets were formatted to the WEKA’s required file format using the FASTA to WEKA converter [28]. The alignment-free datasets were prepared using K-mer frequencies (at k-mer size = 4) using the Logical Alignment Free (LAF) algorithm in Ubuntu OS [55]. Further, in WEKA machine learning, the Support Vector Machine’s (SVM) Sequential Minimal Optimization (SMO) classifier was employed to analyze the aligned and alignment-free datasets through the ten-folds of cross-validation [56]. For the SMO classifier, the filter type used was ‘normalize training data’, which is a process of rescaling one or more attributes to the range of 0 and 1. This parameter can be considered when we do not know the distribution of the data and is present as a default. Then the ‘numFolds’ parameter is for the internal folds for the cross-validation used to generate training data which was kept as ‘-1’, (‘-1’ means use training data) (weka.classifiers.functions.SMO -C 1.0 -L 0.001 -P 1.0E-12 -N 0 -V -1 -W 1 -K). Along with this, polynomial kernel was chosen (weka.classifiers.functions.supportVector.PolyKernel -E 1.0 -C 250007) and calibrator class used was multinomial logistic regression model with a ridge estimator (weka.classifiers.functions.Logistic -R 1.0E-8 -M -1 -num-decimal-places 4). However, those datasets showing lower accuracy or discrimination potential using the Polynomial kernel were then analyzed using the Radial Basis Function, or RBF kernel (weka.classifiers.functions.supportVector.RBFKernel). The polynomial kernel and RBF kernel are simply different in case of making the hyperplane decision boundary between the classes. The Polynomial kernel usually works better with the linear datasets, while the RBF kernel is used when there is no prior knowledge about the data or if the data is in the non-linear form. The RBF kernel was employed by tuning the complexity parameter ‘C’ (Inverse of the strength of regularization) (from 2.0 to 8.0) and the gamma parameter (used only for RBF kernel) (from 0.01 to 0.1) using the WEKA’s object editor. The datasets exhibiting 100% discrimination were further evaluated for overfitting or selection bias by splitting the data into the train set, a subset that is used to train the model, and the test set (unseen data), a subset that is used to test the trained model. Prior to the implementation of the classifier, the dataset was preprocessed by employing the filters from the unsupervised instances. Initially the dataset was randomized, where the order of instances is randomly shuffled with the random seed value (-S) of 42. Further, the dataset was partitioned into the train set using the ‘RemovePercent’ filter (weka.filters.unsupervised.instance.RemovePercentage) and by keeping the ‘InvertSelection’ parameter false, thus removing 30% of the data to create a train set of 70%. Similarly, the test set was created by switching the InvertSelection parameter to true, thus removing the 70% of the data and keeping the 30% of the data as a test set. All the datasets exhibiting 100% discrimination potential were further evaluated using the SMO classifier with the similar parameters that were used previously for cross-validation.

## Results

### PCR amplification and sequencing

**Fig 1:**
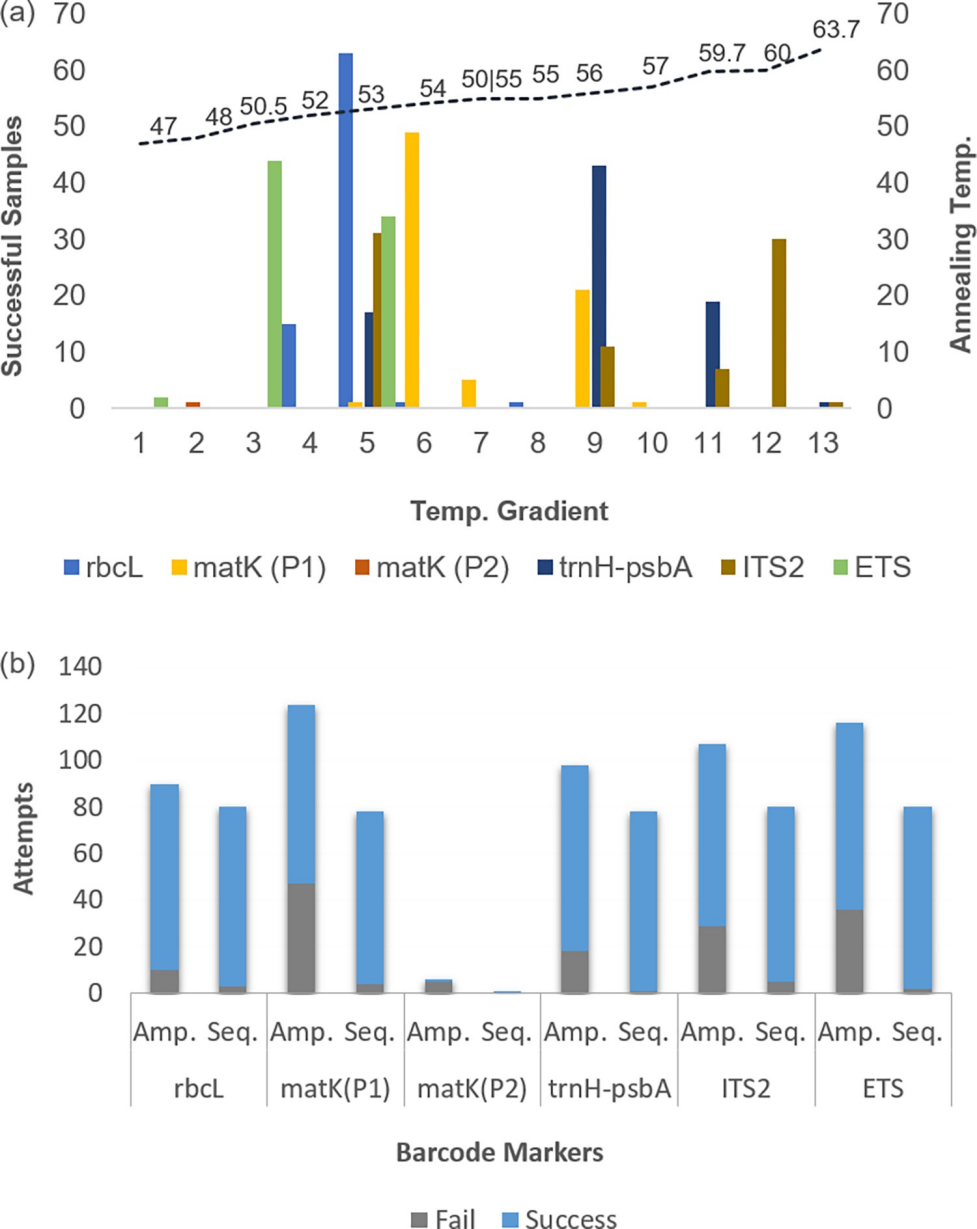
Attempts of PCR amplification and sequencing for the DNA barcode markers employed (a) Annealing temperature gradients employed for amplification, where ’50|55’ indicates an attempt of dual annealing temperature (55 initial and 50 final). (b) Success and failures in the samples attempted for PCR amplification and sequencing.

All of the barcode regions from nrDNAs ETS and ITS2 and cpDNAs rbcL, matK, and trnH-psbA were amplified successfully at various temperature gradients collected from both RAK and UAQ populations (Fig 1(A)).

Almost all markers showed significant success rates (90%-100%) for PCR amplification and sequencing (Fig 1(A) & 1(B)). However, as compared to other primers, matK exhibited the highest rate of failures for the PCR amplification (Fig 1(B)). Thus, additional pair of matK markers (matK (P2) was employed for the amplicon recovery, where only one sample was successfully amplified (Fig 1(A)). Overall, 382 sequences were obtained from 80 specimens of *Salicornia sp*. belonging to the various DNA barcode markers viz., rbcL (n = 77), matK (n = 75), trnH-psbA (n = 77), ITS2 (n = 75) and ETS (n = 78) (Fig 1(B)).

### Taxonomic validation using nrDNA and cpDNA barcode markers

The specimens collected from the RAK and UAQ populations were morphologically identified to the genus level as *Salicornia*. The taxonomic evaluation was further done using the NCBI BLAST. The cpDNA barcodes revealed a multi-species association between 98–100% identity. In trnH-psbA, the barcode identity was less than 97% due to a lack of sufficient DNA barcode sequences from *Salicornia* genera; accessions with the closest match belong to *Salicornia’s* chloroplast genome (*S*. *europaea* (97.67%) (KJ629116.1), *S*. *brachiata* (94.01%) (KJ629115.1) and *S*. *bigelovii* (90.81%) (KJ629117.1)). The nrDNA barcodes even showed multi-species association when subjected to NCBI BLAST. The most relative species resembling the morpho-taxonomy and those recognized in the top search result were *Salicornia persica*, *Salicornia perennans*, and *Salicornia europaea*. However, the correct interpretation of BLAST results requires considerable taxonomic and molecular expertise. Accordingly, we recognized their taxonomic identity close to those three species as identified by nrDNA barcode markers.

**Fig 2:**
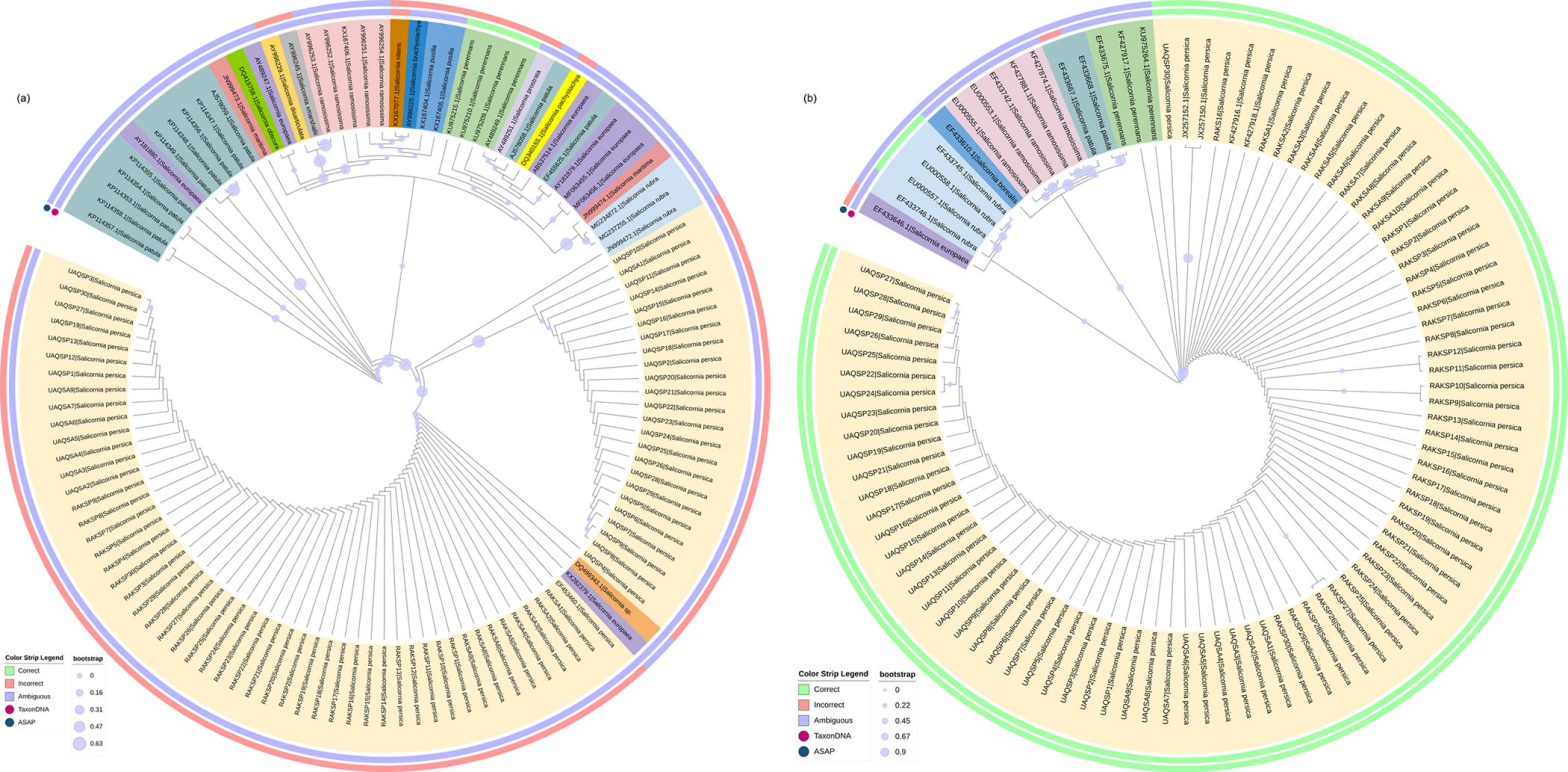
Molecular identification of *Salicornia* sp. using the maximum likelihood approach, and their annotation using OTU picking methods TaxonDNA’s Species identifier and ASAP. ML trees were constructed with bootstrap support (of 1000) and with discrete gamma distribution (a) ML tree of ITS2 sequences obtained using Tamura 3-parameter model, (b) ML tree of ETS sequences obtained using Kimura 2-parameter model.

GenBank’s cpDNA barcode database seems to lack sequences of *S*. *persica* and *S*. *perennans*. However, the ETS region belonging to the nrDNA barcode database appears to have enough sequences than ITS2 as it is one of the well-studied markers in *Salicornia*. From the BLAST results, sequences within the identity of 98–100% belonging to the ITS2 and ETS markers were retrieved. Those sequences were used to construct ML phylogeny by choosing the best suitable model with the 1000 bootstrap support. The tree was further annotated using OTU picking methods for the species resolution using ASAP and TaxonDNA’s Species Identifier (Fig 2).

The ASAP analysis using the ITS2 marker revealed the closest identity to *S*. *persica*, followed by *S*. *europaea* in the form of the merged OTU, though they had only one representative GenBank accession (Fig 2(A)). Moreover, a single GenBank accession DQ499343.1 of the partially identified *Salicornia* sp. exhibited paraphyletic cladding. In TaxonDNA, the studied specimens and GenBank sequences were all grouped, thus representing a merged OTU (Fig 2(A)).

The ETS marker analysis using TaxonDNA exhibited an accurate identification of *S*. *persica* (for all specimens) at a 3% threshold (Fig 2(B)). Similarly, the ASAP analysis showed a correct match to *S*. *persica* at the third partition (Threshold distance = 0.002964) using JC69 distance metrics (Fig 2(B)). Altogether, the morpho-taxonomic and nrDNA barcode analysis using the OTU picking methods, the TaxonDNA, and ASAP reveal that the specimens collected from RAK and UAQ populations belong to the species *Salicornia persica*.

### Genetic divergence between RAK and UAQ populations

The sequences belonging to the RAK and UAQ sampling sites were analyzed for genetic divergence using the alignment-based and alignment-free unsupervised OTU picking and supervised machine learning approach.

The analysis was done for every individual marker and the concatenated multilocus markers. Overall, three concatenated barcode datasets were created, viz. the cpDNA (rbcL, matK, and trnH-psbA), nrDNA (ITS2 and ETS), and cpDNA + nrDNA (rbcL, matK, trnH-psbA, ITS2 and ETS). The concatenation was done to analyze the discrimination potential of multilocus barcode markers when employed together.

The assessment through the alignment-based unsupervised OTU picking method using TaxonDNA revealed the highest rate of population discrimination of 96% for matK followed by cpDNA datasets (72%) (Table 1). The incorrect identification was observed for RAKSA1 and RAKSP14, while the ambiguous match recognized through the Best Match and Best Closest Match criteria was UAQSP10. Similarly, ASAP showed the highest rate of population differentiation of 98.66% for matK and cpDNA, respectively (Table 1). However, a split in the UAQ group was observed using JC69 and K80 metrics at a threshold distance of 0.000594 (ASAP score = 2.5), where UQSA1 was split into the third group.

**Table 1:** Discrimination potential of multilocus barcode markers to differentiate RAK and UAQ populations using unsupervised and supervised learning approach.

Approach	Tool (dataset)	Metric	cpDNA+ nrDNA	cpDNA	nrDNA	rbcL	matK	psbA-trnH	ITS2	ETS
Unsupervised Learning	TaxonDNA (AL)	Best match & Best Closest match (Kimura-2-parameter)	66.66	72	37.83	0	96	0	30.66	3.84
	ASAP (AL)	Jukes-Cantor (JC69)	29.16	98.66	32.43	NA	98.66	NA	30.66	0
	CAFE (AF)	Co-phylog	100	100	37.83	NA	100	NA	30.66	12.82
Supervised Learning	WEKA (AL)	Sequential minimal optimization (SMO)	100^a^	100^a^	81.08^a^	71.42^a^	100^a^	50.64^a^	80^a^	60.25^a^
	WEKA (AF)		100^a^	100^a^	78.37^b^	74.02^a^	100^a^	55.84^c^	80^c^	66.66^c^

Abbrevations: AL = Alignment, AF = Alignment free, NA = Not available; Annotations

^a^ = Polykernel

^b^ = RBFkernel with -C 8.0 -G 0.01

^c^ = RBFkernel with -C 8.0 -G 0.1.

**Fig 3:**
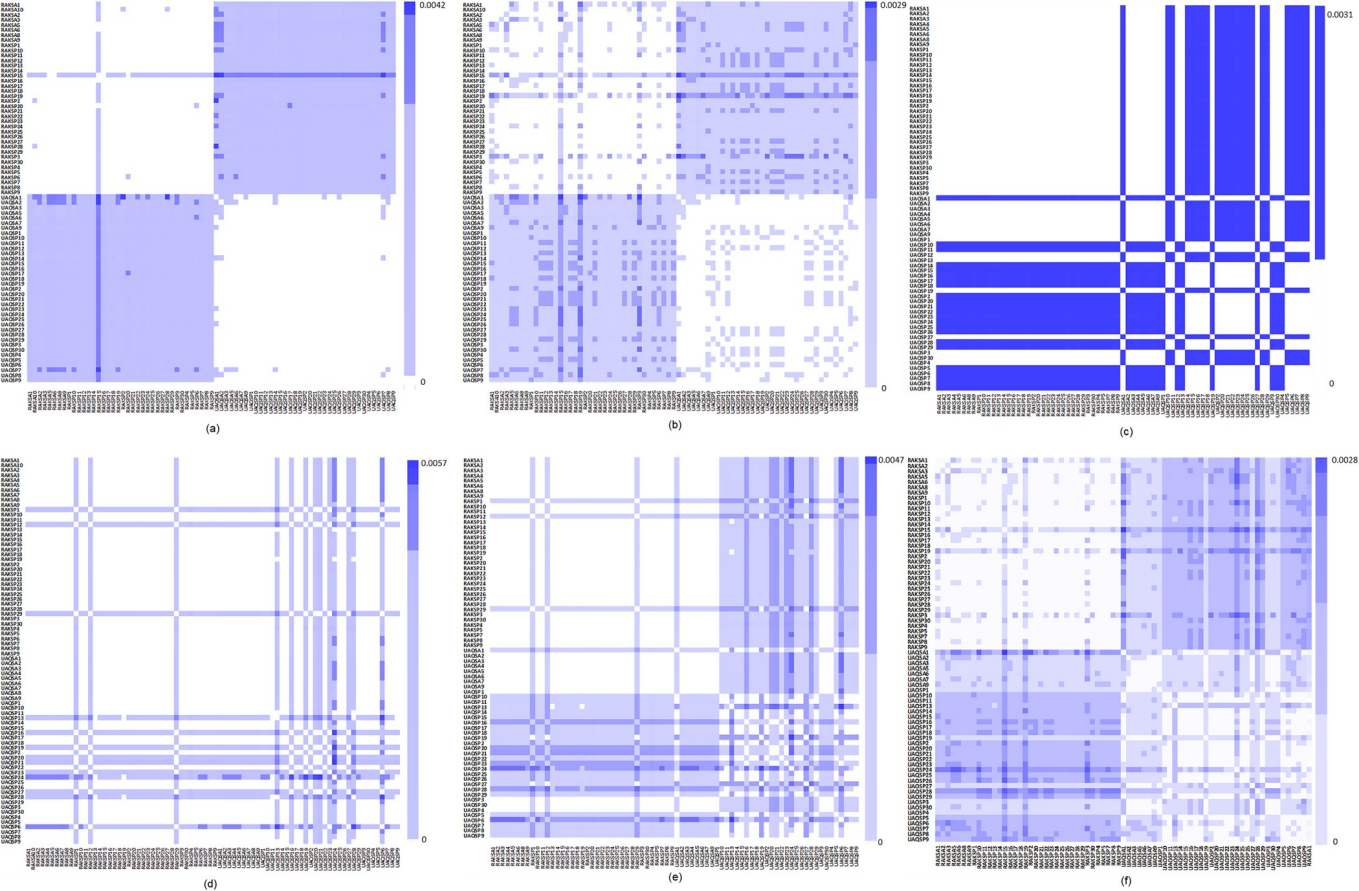
Heat map showing genetic divergence between populations (RAK & UAQ) in *Salicornia persica* using alignment-free approach (Co-Phylog). (a) matK (b) cpDNA (c) ITS2 (d) ETS (e) nrDNA (f) cpDNA+nrDNA.

The alignment-free Co-phylog method revealed no population differentiation using s plastid DNA barcode markers the rbcL and psbA-trnH when employed individually. However, significant discrimination between the two populations, RAK and UAQ, was observed using the matK marker (Fig 3(A)) (Table 1). Moreover, when all the studied plastid barcode markers were analyzed together (as a cpDNA dataset), both the populations exhibited proper discrimination (Fig 3(B)). For the nrDNA barcode markers ITS2 and ETS, no significant population differentiation was observed when employed individually (Fig 3(C) & 3(D)). However, the concatenated dataset of nrDNA barcode markers revealed better results, though splitting of the UAQ population was observed for twelve individuals (UAQSA2 to UAQSA7, UAQSA9, UAQSP1, UAQSP3, UAQSP4, UAQSP13, and UAQSP30) which were merged with the RAK population (Fig 3(E)). Further, the co-phylog distances were analyzed through ASAP to determine the population groups. Overall, four groups were recognized, of which the first group represented the merge of twelve individuals as above, thus limiting the accuracy of nrDNA to only 37.83% (Table 1). However, when all markers were concatenated (cpDNA + nrDNA), both the populations were resolved successfully (Fig 3(F)) (Table 1).

**Fig 4:**
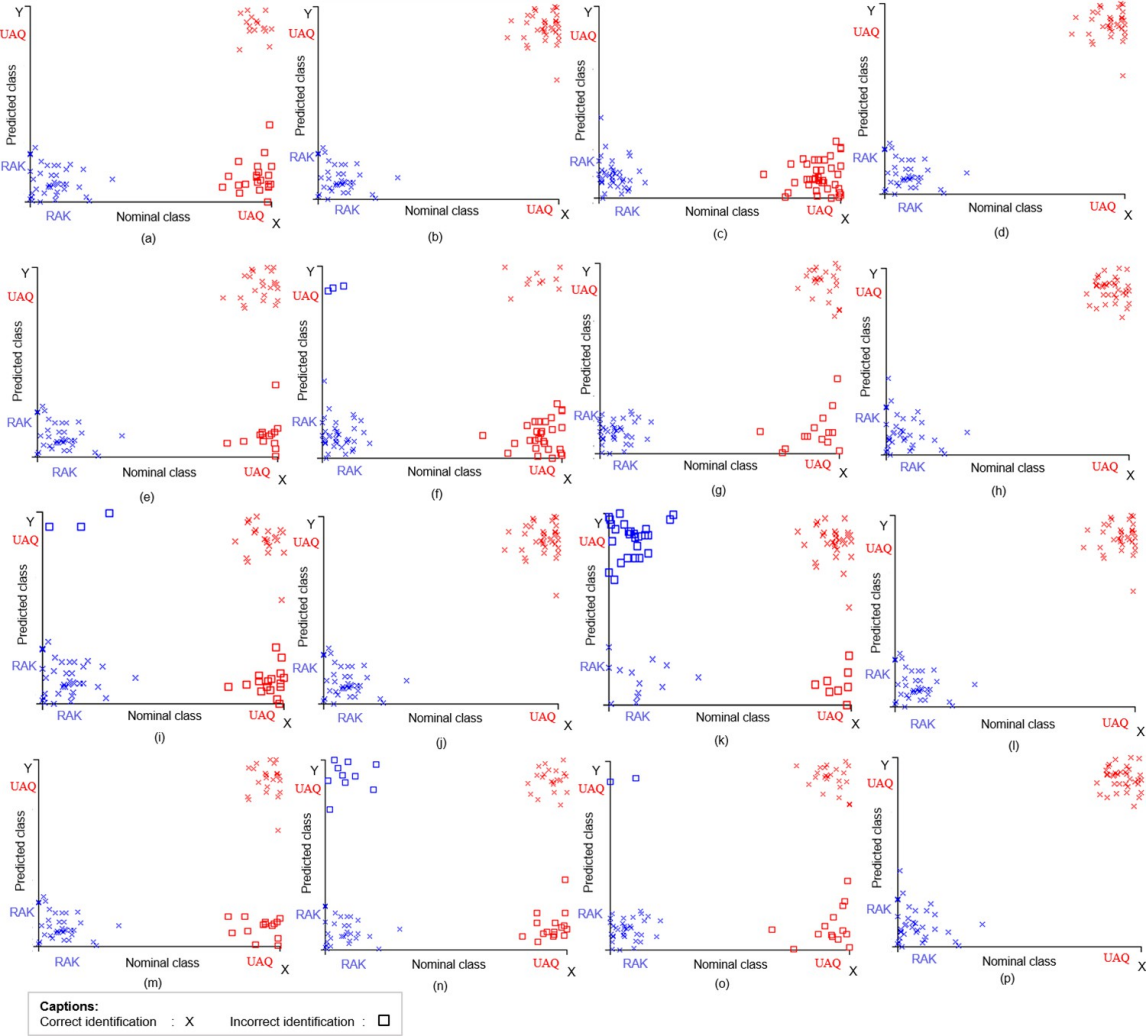
Scatter plot representing the nominal and predicted distribution of the individuals across the sampling sites (RAK and UAQ). (I) Alignment based datasets analyzed using SML’s SMO classifier (a) rbcL (b) matK (c) psbA-trnH (d) cpDNA (e) ITS2 (f) ETS (g) nrDNA (h) cpDNA+nrDNA. II) Alignment free datasets analyzed using SML’s SMO classifier (i) rbcL (j) matK (k) psbA-trnH (l) cpDNA (m) ITS2 (n) ETS (o) nrDNA (p) cpDNA+nrDNA.

Using the alignment-based and alignment-free approach, the SML method with the supervised vector machine’s SMO (Sequential Minimal Optimizer) classifier resolved both the populations with 100% efficiency (for the matK, cpDNA, and cpDNA+nrDNA datasets) using the Polynomial kernel (Fig 4(B), 4(D) & 4(H)). However, the other AL and AF datasets (rbcL, psbA, ITS2, ETS, and nrDNA) exhibiting lower resolution potential were further evaluated using the SMOs RBF kernel against the Polynomial kernel (S1 Fig). It was observed that those AL datasets (nrDNA, ITS2, ETS, rbcL & psbA-trnH) exhibited higher resolution potential using the Polynomial kernel. However, almost all the selected unaligned datasets excluding rbcL gave better results using the RBF kernel than the Polynomial kernel, given the complexity parameter ‘C’ (Inverse of the strength of regularization) and the gamma parameter (used only for RBF kernel) were tuned using the WEKA’s object editor (S1 Fig). Though the datasets other than the constituents of matK have exhibited lower resolution potential, most AF datasets have shown higher efficiency (Table 1). Datasets rbcL, psbA, ITS2, ETS, and nrDNA demonstrated lower efficiency but a higher resolution potential than the unsupervised methods (Table 1).

Overall, the alignment-free approach (Fig 4(I)–4(O)) stands out to be most competent than the alignment-based approach (Fig 4(A)–4(H)) for all other markers and concatenated datasets except for the nrDNA dataset (Fig 4(G) & 4(O)), where the alignment-based approach showed a higher rate of population discrimination (Table 1).

Taken together, it seems that the matK marker tends to exhibit higher discrimination potential as compared to the other barcode markers when analysed individually or in the concatenated datasets. Wherever the matK barcode marker was concatenated (cpDNA and cpDNA+nrDNA), significant differentiation was observed between the populations of *S*. *persica* (up to 100%) (Table 1). Following the success of matK dataset, the nrDNA dataset exhibited a higher rate of population discrimination of 81.08% (SML(AL)).

However, the observed 100% discrimination for the matK, cpDNA, and cpDNA+nrDNA datasets using the train set could lead to overfitting or selection bias. To rule out the overfitting of the selected model, we evaluated the datasets by splitting them into a train set (70%) to train the model and a test set (30%) to test the trained model. The results were similar to our previous observations, where all the selected datasets exhibited 100% accuracy for the chosen classifier (SMO). Thus discriminating both the populations with the 100% accuracy.

**Fig 5:**
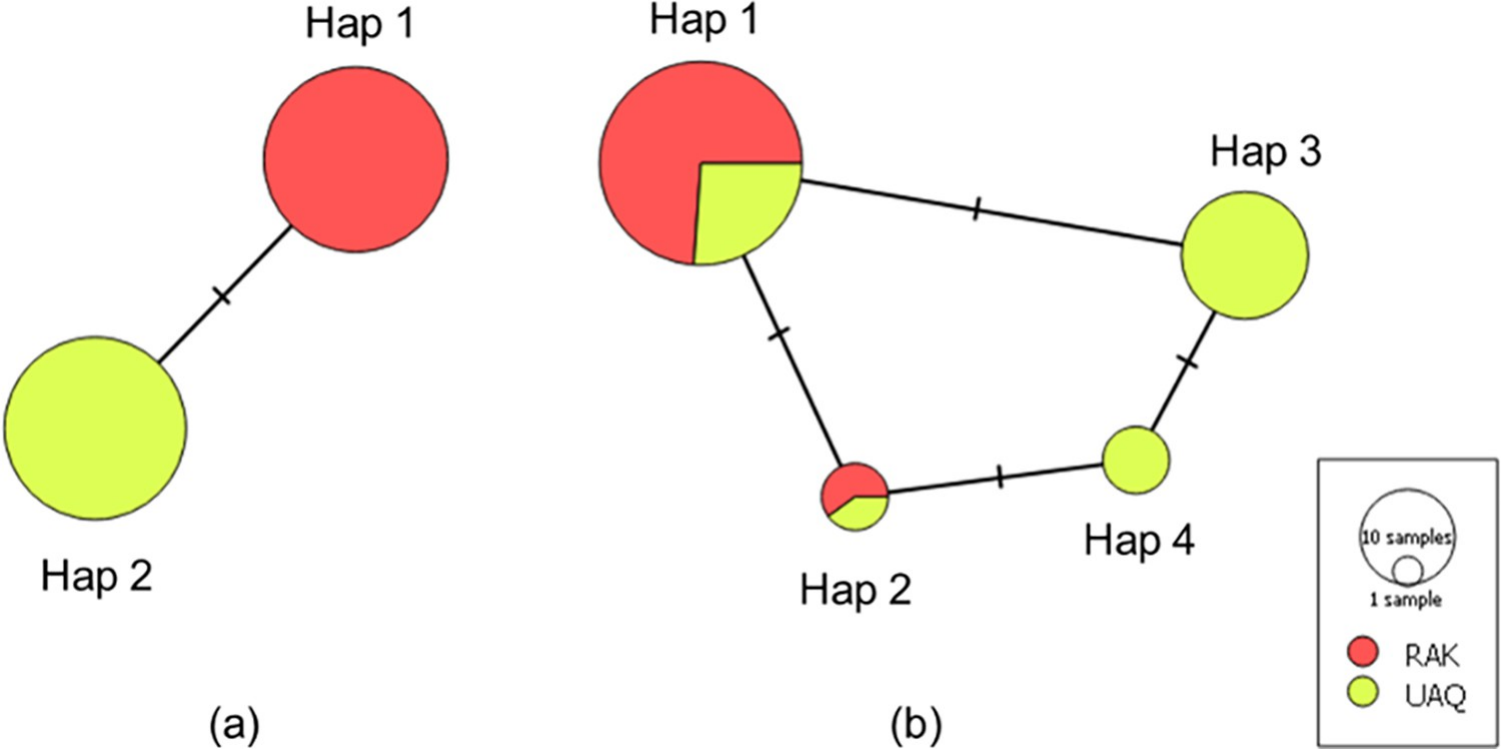
Minimum spanning network representing *Salicornia persica* haplotypes belonging to Ras-Al-Khaimah and Umm-Al-Quwain populations. (a) matK haplotype network (b) nrDNA (ITS2+ETS) haplotype network.

Furthermore, both datasets (matK and nrDNA) were analysed for the genetic divergence within and between the populations. The matK dataset revealed haplotype diversity of 0.506 with one variable site. The nucleotide diversity (π) of zero was observed within both the populations RAK and UAQ respectively, which is obvious as both the populations possess homologous sequences that are distinctly differentiated with just one mutation and a polymorphic site (Fig 5(A)). The nrDNA dataset showed haplotype diversity of 0.552 and nucleotide diversity (π) of 0.00017 for RAK and 0.00087 for UAQ populations with one and two polymorphic sites, respectively. Moreover, Hap 1 and Hap 2 showed mixed haplotype distribution with individuals from RAK and UAQ populations (Table 2), while Hap 3 and Hap 4 represented individuals exclusively from the UAQ population only (Fig 5(B)) (Table 2).

**Table 2:** Haplotype distribution of *Salicornia persica* belonging to matK and nrDNA datasets.

Datasets	Total haplotypes	Hd	Haplo-types	Number of Individuals	Haplotype composition
matK	2	0.5067	Hap 1	38	RAKSA1—RAKSA3, RAKSA5, RAKSA6, RAKSA8—RAKSA10, RAKSP1—RAKSP30
			Hap 2	37	UAQSA1—UAQSA3, UAQSA5—UAQSA7, UAQSA9, UAQSP1—UAQSP30
nrDNA	4	0.5528	Hap 1	46	RAKSA1—RAKSA6, RAKSA8, RAKSA9, RAKSP2—RAKSP5, RAKSP7—RAKSP11, RAKSP13—RAKSP28, RAKSP30, UAQSA2—UAQSA7, UAQSA9, UAQSP1, UAQSP3, UAQSP4, UAQSP13, UAQSP30
			Hap 2	5	RAKSP1, RAKSP12, RAKSP29, UAQSP19, UAQSP27
			Hap 3	18	UAQSA1, UAQSP2, UAQSP5, UAQSP7—UAQSP11, UAQSP14, UAQSP15, UAQSP17, UAQSP18, UAQSP22, UAQSP24—UAQSP26, UAQSP28, UAQSP29
			Hap 4	5	UAQSP16, UAQSP20, UAQSP21, UAQSP23, UAQSP6

Altogether, the alignment and alignment-free approaches using the unsupervised and supervised learning methods efficiently demonstrated the matK marker to discriminate individuals from the RAK and UAQ populations. Here, the SML’s AF approach exhibits the highest discrimination potential compared to the other methods. Moreover, the haplotype analysis revealed clear population distinction using the matK marker; though, the nrDNA dataset (ETS & ITS2) showed much higher variance than observed morphologically.

## Discussion

Due to the well-known variable morphology of the genus *Salicornia*, researchers worldwide have used many different species interpretations. This study represents the first attempt to utilize multilocus markers to detect genetic diversity and species discrimination in the *Salicornia* populations in UAE. For such purpose, the combined multiple markers approach has been considered more powerful in detecting genetic variation among plant species [8, 9, 57, 58]. In the present study, we identified the *Salicornia* species in UAE as *S*. *persica* using the ETS and ITS2 markers.

Moreover, the genetic divergence analysis shows that the RAK population is different from the population of UAQ. Plants from both populations occupy almost similar habitats as typical halophytes; they are found in coastal salt marshes, tidal mudflats, tidal oscillation zones, and the edge of lagoons and are always associated with high saline habitats. Based on the analysis, the UAQ population is undoubtedly differentiated from the RAK; however, determining the reason behind the deep divergence would require concrete supportive morphological and taxonomic studies.

Literature suggests that Eurasian *Salicornia’s* tax*a* are often divided into two species or species groups as per the standard floras and checklists [59, 60]. This division has been done based on their ploidy levels as the diploid *S*. *europaea* group, and tetraploid *S*. *procumbens* group contain several microspecies. The species concept was ultimately challenged within these two by molecular analyses, where *S*. *procumbens*, *S*. *persica* tend to exhibit monophyla and are hence regarded as cryptic species. Moreover, both phylogenetic [20] and population genetic [61] data could indicate that the difference between their habitats rather than morphology accounts for the observed patterns of genetic variation.

Such observations called for the extensive molecular assessment for recognizing the genetic variation in the *Salicornia* sp. collected from the two populations of RAK and UAQ using DNA barcode markers individually (plastid markers: rbcL, matK and trnH-psbA and nuclear marker: ITS2 and ETS region) as well as with a combination of multilocus markers. It has been proven that multiple markers are more powerful in detecting genetic variation among plant species [8, 9, 17, 57, 58].

The effectiveness of markers in species resolution was assessed initially by verifying the sequence identity using NCBI BLAST. Multiple species were observed within 3% identity for most barcode markers (excluding ETS), thus forming a monophyletic clade. Similarly, Manton [62] and Kadereit et al. [20] observed monophyletic cladding for the *Salicornia* genera. However, the ETS region is a well-established marker for characterizing the *Salicornia* group to define and recognize species, subspecies, and genotypes [20]. Thus, with the potential of the ETS marker and assistance of OTU picking methods, we recognized *Salicornia* sp. from studied populations as *S*. *persica*, previously considered *S*. *europaea* [37–40]. Similarly, the recent re-examination of *Salicornia* in Saudi Arabia reveals the presence of *S*. *persica* and *S*. *sinus*-*persica*; as before, it was considered *S*. *europaea* [63].

Moreover, Shahid et al. [38] have reported phenotypic variation in *Salicornia* populations of RAK and UAQ and suggested two different ecotypes of the species they considered *S*. *europaea*. Similarly, our field observations for the RAK and UAQ populations suggest that the *Salicornia* genus might represent two independent lineages or a closely related species. Therefore, multilocus barcode markers were employed to elucidate our suspicion of the presence of more than one species closer to the species confirmed in this study as *S*. *persica*.

Results reveal a non-monophyletic relationship (for matK and nrDNA datasets) between the populations by using the alignment-free tool CAFÉ (Co-phylog metric) and advanced alignment & alignment-free SML technique (SVM algorithm). The Co-phylog and SVM successfully discriminate (100%) UAQ and RAK specimens using the matK marker (Table 1). The alignment-free Co-phylog algorithm has previously demonstrated its efficiency and delivers high resolution and accurate phylogenies of closely related species [24, 27]. Moreover, the supervised learning method was employed, which outperforms other approaches. Likewise, machine learning algorithms have demonstrated their effectiveness in resolving plant taxa [28–31, 64]. Higher genetic divergence was observed using the nrDNA (ETS+ITS2) dataset exhibiting four haplotypes in the studied populations. The nrDNA has always shown higher genetic divergence than the cpDNA [65, 66]. Indeed, the cpDNA tends to evolve very slowly, with low recombination and mutation rates [67]. However, the cpDNA lineages usually show the unique geographical distribution and evolutionary history of natural populations and therefore have been widely used [68]. In our study, the cpDNA’s matK marker has shown a higher rate of population discrimination (100%) with two distinct homogenous haplotypes representing RAK and UAQ populations, respectively. Manton [62] recorded similar observations for the *Salicornia* genera using matK and rbcL+matK markers.

Other cpDNA markers, viz., rbcL and psbA-trnH are more convenient in amplification, sequencing, and aligning; though matK is difficult to amplify, it shows excellent discriminatory power (CBOL, 2009 [15]) (Fig 1, Table 1). We obtained high-quality barcodes for rbcL and psbA-trnH, but they could not show significant discrimination, unlike matK, which showed higher resolution [15, 69, 70].

Altogether, the matK marker was significantly able to discriminate UAQ and RAK populations, indicating the existence of a genetically diverged species or different morphotypes (Fig 5).

## Conclusion

The earlier studies dealt with UAE *Salicornia* as the European species, *S*. *europaea*. However, based on field observations and the evidence from the NCBI BLAST of ETS barcodes supported by extensive analysis through OTU picking methods, the identity of *Salicornia* from RAK and UAQ populations was confirmed as *S*. *persica*. Moreover, the matK marker significantly differentiated the two populations using the alignment-free Co-Phylog technique and the alignment and alignment-free supervised machine learning approach. Further investigations focusing on the plants’ morpho-taxonomic characterization are in progress to get better insights towards determining the morph-types in both the population of *Salicornia* in UAE.

## Supporting information

S1 Table List of primers used in this study. (DOCX)

S1 Fig Evaluation of (a) Aligned and (b) Alignment-free datasets using the Polykernel (C: 1.0) and RBF kernel (C: 2.0 to 8.0 and G: 0.01 as default except ‘RBF -C 8.0 –G 0.1’ where G is 0.1). Abbreviations: ‘C’ is complexity parameter, and ‘G’ is gamma parameter used only for RBF kernel.
(TIF)
