# Supplementary material for: Multilocus marker-based delimitation of Salicornia persica and its population discrimination assisted by supervised machine learning approach
Source: PLoS One. 2022 Jul 27;17(7):e0270463. doi: 10.1371/journal.pone.0270463 (PMC9328517; doi:10.1371/journal.pone.0270463)
Supplement: S1 Table — (DOCX) [file pone.0270463.s001.docx]

**Table S1. List of Primers used in this study.**

| **Locus** |  | **Primer Id** | **Sequence (5' to 3')** |
| --- | --- | --- | --- |
| rbcL |  | rbcLa-F | ATGTCACCACAAACAGAGACTAAAGC |
|  |  | rbcLa-R | GTAAAATCAAGTCCACCRCG |
| matK | P1 | MatK-1RKIM-f | ACCCAGTCCATCTGGAAATCTTGGTTC |
|  |  | MatK-3FKIM-r | CGTACAGTACTTTTGTGTTTACGAG |
|  | P2 | MatK_390f | CGATCTATTCATTCAATATTTC |
|  |  | MatK_1326r | TCTAGCACACGAAAGTCGAAGT |
| psbA-trnH |  | psbA3_f | GTTATGCATGAACGTAATGCTC |
|  |  | trnHf_05 | CGCGCATGGTGGATTCACAATCC |
| ITS2 |  | ITS2-S2F | ATGCGATACTTGGTGTGAAT |
|  |  | ITS4 | TCCTCCGCTTATTGATATGC |
| ETS |  | Salicornia-F | GTCCCTATTGTGTAGATTTCAT |
|  |  | 18S-II | CTCTAACTGATTTAATGAGCCATTCGCA |
