# Supplementary figures and images for: Multilocus marker-based delimitation of Salicornia persica and its population discrimination assisted by supervised machine learning approach
Source: PLoS One. 2022 Jul 27;17(7):e0270463. doi: 10.1371/journal.pone.0270463 (PMC9328517; doi:10.1371/journal.pone.0270463)

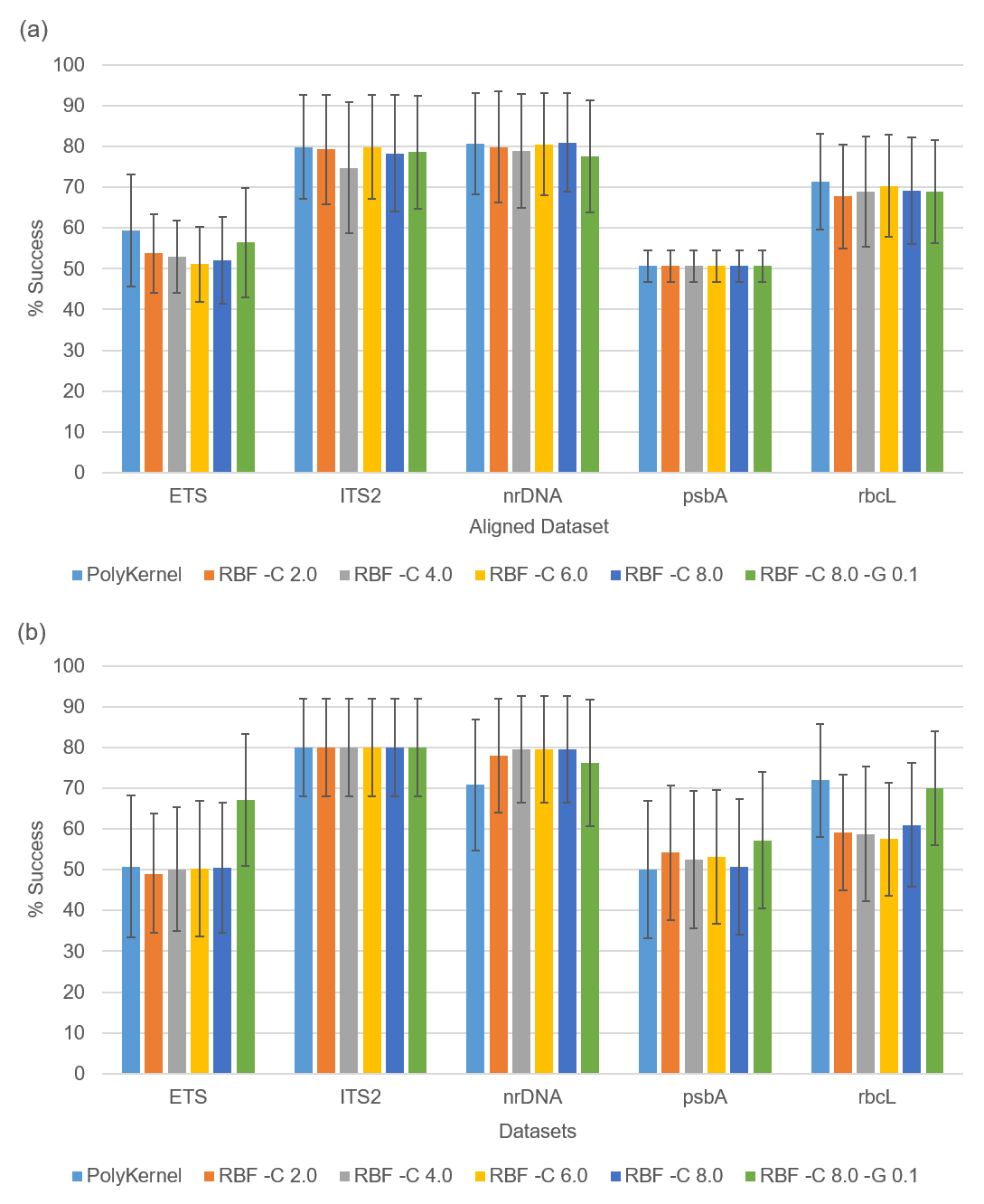

Supplement: S1 Fig — Evaluation of (a) Aligned and (b) Alignment-free datasets using the Polykernel (C: 1.0) and RBF kernel (C: 2.0 to 8.0 and G: 0.01 as default except ‘RBF -C 8.0 –G 0.1’ where G is 0.1). Abbreviations: ‘C’ is complexity parameter, and ‘G’ is gamma parameter used only for RBF kernel. (TIF) [file pone.0270463.s002.tif]
